# Sex-based influential factors for dental caries in patients with schizophrenia

**DOI:** 10.1186/s12888-023-05256-z

**Published:** 2023-10-10

**Authors:** Mi Yang, Jingjing Xu, Xiaoqin Chen, Liju Liu, Di Kong, Yan Yang, Wei Chen, Zezhi Li, Xiangyang Zhang

**Affiliations:** 1 Department of Psychiatry, The Fourth People’s Hospital of Chengdu, No.8 Huli-West 1st-Alley, Jinniu District, Chengdu, 610036 China; 2 MOE Key Lab for Neuroinformation, The Clinical Hospital of Chengdu Brain Science Institute, University of Electronic Science and Technology of China, Qingshuihe Campus: No.2006, Xiyuan Ave, West Hi-Tech Zone, Chengdu, 611731 China; 3 School of Life Science and Technology, University of Electronic Science and Technology of China, Qingshuihe Campus: No.2006, Xiyuan Ave, West Hi-Tech Zone, Chengdu, 611731 China; 4 Department of Psychiatry, Qingdao mental health center, No. 299, Nanjing Road, Qingdao, 266034 China; 5 Department of Nutritional and Metabolic Psychiatry, The Affiliated Brain Hospital of Guangzhou Medical University, 36 Mingxin Road, Liwan District, Guangzhou, 510370 China; 6 Department of Psychiatry, Guangdong Engineering Technology Research Center for Translational Medicine of Mental Disorders, 36 Mingxin Road, Liwan District, Guangzhou, 510370 China; 7 Key Laboratory of Neurogenetics and Channelopathies of Guangdong Province, Ministry of Education of China, Guangzhou Medical University, 36 Mingxin Road, Liwan District, Guangzhou, 510370 China; 8 CAS Key Laboratory of Mental Health, Institute of Psychology, Chinese Academy of Sciences, 16 Lincui Road, Chaoyang District, Beijing, 100101 China

**Keywords:** Sex difference, Schizophrenia, Caries, Cognition, Diabetes, Smoking

## Abstract

**Background:**

Schizophrenia is a common mental disorder that seriously affects patients’ daily lives and brings heavy psychological and economic burdens to their families and society. The oral problems of patients with schizophrenia are gradually gaining attention, among which dental caries are among the most common oral diseases. Sex differences may be related not only to the various clinical symptoms of schizophrenia but also to different oral hygiene statuses; therefore, the main purpose of this paper is to investigate sex differences related to influencing factors for dental caries in patients with schizophrenia.

**Method:**

Inpatients with schizophrenia over 18 years old were included in this study, and multidimensional indicators such as demographics, symptom and cognitive impairment assessments, medications, and the caries index of decayed, missing, and filled teeth (DMFT) were collected. An analysis of sex-based influential factors for dental caries in schizophrenia patients was performed.

**Results:**

Four-hundred and ninety-six patients with schizophrenia were included, with a mean age of 46.73 ± 12.23 years, of which 142 were females and 354 were males. The mean DMFT was significantly higher in males (8.81 ± 8.50) than in females (5.63 ± 6.61, p < 0.001), and the odd ratio of caries in males to females was significantly higher as well (OR = 2.305, p < 0.001). The influential factors of caries in male patients were independently associated with age and smoking status, in which current smokers were at the highest risk for developing caries, and different smoking statuses had various influencing factors for caries. The influencing factors for caries in female patients were independently associated with age, antipsychotic dose, PANSS-positive symptoms, and MMSE levels.

**Conclusion:**

Our findings suggest sex differences exist among influential factors for caries in patients with schizophrenia. These risk factors may even be associated with and affect the treatment and prognosis of psychiatric symptoms in patients. Therefore, oral hygiene management of patients with schizophrenia should be enhanced. These differential factors provide new visions and ideas for formulating individual interventions, treatments, and care priorities.

**Supplementary Information:**

The online version contains supplementary material available at 10.1186/s12888-023-05256-z.

## Introduction

Schizophrenia is a common serious psychiatric disorder characterized by positive symptoms, negative symptoms, and cognitive deficits [1] and imposes a heavy psychological and economic burden on families, households, and society [2, 3]. Sex differences in symptoms, cognition, medication preferences, and prognosis are currently present in patients with schizophrenia. For example, men have stronger objective social cognition correlated with nonsocial functioning [4] and visual working memory function [5] than women. Meanwhile, women have verbal fluency associated with hostile bias [6] and perform better than men in processing speed, switching, and verbal situational memory [5]. Sex-based differences in subjective tolerance of antipsychotic medications [7] lead to better medication efficacy and prognosis in women than men [8], but women are more likely to gain weight [9]. These differences may be related to the differences in brain structure and brain function [10], gene expression [11], or the microbiota-brain-gut axis [12].

Dental caries is the most common oral disease. Caries can affect the chewing function of patients and the growth and development of affected children [13] and is the leading cause of tooth loss, with an increasing trend in the rate of untreated dental caries [14, 15]. Some studies have found that dental caries can reflect the change in the inorganic level of the human body [16], the degree of socioeconomic stress [17], and the degree of depression and anxiety of patients [18]. The development of dental caries is often associated with smoking, genetics, dietary preferences, inadequate salivary secretion, poorly controlled diabetes [19], or oral flora disorders [20]. Studies have shown that the dominant oral microbial flora causing caries differs from periodontal and gingival diseases [21]. There are controversies regarding sex differences in caries occurrence. Large-scale epidemiological surveys in South Asia [22], China [23, 24], Portugal [25], or Russia [26] have shown that women are more likely to develop caries, which may be explained by a higher intake of snacks than men [27], hormonal fluctuations due to pregnancy or menstruation [27], or a greater abundance of acid-producing cariogenic *lactococci* [28]. However, among hospitalized patients with severe schizophrenia, male elderly patients have poorer oral hygiene than females, including a higher caries index and risk [29, 30].

There is still a lack of comprehensive studies on sex differences in caries and related multidimensional factors in patients with schizophrenia. Therefore, this paper intends to investigate the influential factors for sex-based differences in caries in patients with schizophrenia, to find the clinical representation and theoretical basis of oral microbial community differences, and to help develop a comprehensive strategy for sex-based differential oral management in patients with schizophrenia.

## Methods

The Beijing Huilongguan Hospital Ethics Committee approved the study, and all patients or their legal guardians provided written informed consent. The protocol involving human participants and human data has been performed in accordance with the Declaration of Helsinki. This reporting was performed per the Strengthening the Reporting of Observational studies in Epidemiology (STROBE) guidance [31].

### Patients

Patients with schizophrenia who met the inclusion criteria from four psychiatric hospitals were included in this study. We collected patient demographics (age, sex, BMI), substance dependence status (smoking, drinking), comorbidities (diabetes, hypertension, suicide status, insomnia status), psychiatric history, medications (type of medication, dose), Positive And Negative Syndrome Scale (PANSS) [32], Mini-Mental State Examination (MMSE), Global Deterioration Scale (GDS) [33], Repeatable Battery for the Assessment of Neuropsychological Status (RBANS) [34] and oral health status.

Inclusion criteria: 1, Han Chinese inpatients aged 18 years and over; 2, patients with schizophrenia diagnosed by the Structured Clinical Interview for the DSM-IV (SCID) criteria. Exclusion criteria: 1, patients with epilepsy, cranial injury, periodic paralysis, or other neurological disorders; 2, patients with cardiovascular disease, other metabolic disorders, or endocrine disorders; 3, patients with substance dependence (not tobacco or alcohol dependence); 4, patients who were unable to cooperate with the completion of oral and all other tests; 5, patients with incomplete data.

### Caries diagnosis

The DMFT (decayed, missing, and filled teeth) is a commonly used caries assessment index that reflects oral hygiene status [35]. According to the World Health Organization’s oral hygiene standards [36], caries is defined as “a lesion in a pit or fissure, or on a smooth tooth surface, [that] has an unmistakable cavity, undermined enamel, or a detectably softened floor or wall.” The decayed tooth (DT), missing tooth (MT), and filled tooth (FT) counts of the patients were measured with an orofacial microscope and probe, and the cases of tooth loss and filling due to non-carious (traumatic) factors were excluded. The number of 28 permanent teeth was used as the reference for the number of teeth in a healthy Chinese adult [37]. DMFT > 0 was used as the grouping condition for caries and non-caries. Dentists were trained uniformly before the assessment and passed the kappa concordance test (κ = 0.90).

### Statistical analysis

Statistical analysis was performed using SPSS 26 (IBM Corporation, New Orchard Road, Armonk, NY 10,504, USA). Categorical variables were analyzed by the chi-square test. Continuous variables were first tested for normality by the Kolmogorov-Smirnov test and then for homogeneity of variance by the Levene test. The Mann-Whitney U-test analyzed non-normality or variance-inequality variables. Normal variables were tested by *t*-test. With covariates including age, BMI, smoking, and illness duration, a two-way analysis of covariance (ANCOVA) was used for sex differences in clinical symptoms and medication with independent predictors being sex (male vs. female) and diagnosis (caries vs. non-caries). Correlation analysis was performed using the Spearman test, binary logistic regression was used for independent factor analysis of caries, and the Bonferroni Test was used for multiple comparison correction (*α* = 0.05/35 ≈ 0.0014). Continuous variables were shown as mean ± standard deviation ($\stackrel{\text{-}}{\text{x}}\pm\text{ std.}$). A p ≤ 0.05 was considered with a statistical significance.

## Results

### Participant demographics

After excluding those who did not meet the inclusion criteria from 988 investigated patients, 496 patients were included for the analysis, with a mean age of 46.73 ± 12.23 years and a mean duration of illness of 21.42 ± 11.81 years, of whom 142 were women and 354 were men. Table 1 shows the significant differences between men and women in terms of DMFT, DT, caries status, BMI, marriage, education levels, smoking, drinking, diabetes, family history, age at first hospitalization, types and numbers of antipsychotic medications, and GDS ranks (all p < 0.05). Only DMFT, DT, caries status, marital status, smoking, drinking, and family history passed the Bonferroni test (*α ≈* 0.0014). Oral problems, including DMFT, DT, and caries risk (odd ratio, OR = 2.305), were more significantly severe in men than in women (all p < 0.05). Table 2 shows an interaction effect on age, education, and age at first hospitalization between caries and sex. Only the interaction effect of age with sex (p < 0.001) was Bonferroni corrected (*α* = 0.0014).

**Table 1:** Characters between female and male patients with schizophrenia

	Female (n = 142)	Male (n = 354)	*Z, t or χ* ^*2*^	*p*
**DMFT**	5.63 ± 6.61	8.81 ± 8.50	−4.093	< 0.001
DT	2.12 ± 3.13	4.42 ± 5.46	−4.359	< 0.001
MT	2.80 ± 4.78	3.92 ± 6.42	−1.186	0.236
FT	0.72 ± 3.07	0.47 ± 1.57	−0.911	0.362
**Caries status** (No/Yes)	35/107	44/310	11.299	0.001
**Age** (years)	45.58 ± 12.07	47.19 ± 12.28	−1.196	0.232
**BMI** (kg•m^− 2^)	25.18 ± 4.44	24.23 ± 3.67	−1.990	0.047
**Marriage** (Unmarried/Married/Divorced/Widowed)	64/47/25/6	236/60/56/2	28.684	< 0.001
**Education** (Primary/Junior/Senior/Bachelor)	10/58/49/25	46/160/113/35	8.816	0.032
**Smoking** (Never/Used/Now)	132/9/1	137/67/150	123.294	< 0.001
**Drinking** (No/Yes)	132/10	242/112	33.058	< 0.001
**Hypertension** (No/Yes)	120/22	309/45	0.671	0.413
**Diabetes Mellitus** (No/Yes)	112/30	309/45	5.591	0.018
**Family History** (No/Yes)	105/37	308/46	12.410	< 0.001
**First-Episode Onset Age** (years)	22.88 ± 7.73	25.49 ± 7.57	−1.006	0.314
**First Hospitalization Age** (years)	26.30 ± 6.89	29.27 ± 10.29	−2.738	0.006
**Illness Duration** (years)	20.70 ± 11.91	21.71 ± 11.78	−0.856	0.392
**Antipsychotics Dosage** (mg/day)	555.83 ± 1255.40	367.80 ± 418.57	−0.592	0.554
**Antipsychotics Type** (Typical/Atypical/Both)	2/127/13	0/340/14	10.048	0.007
**Antipsychotics Numbers** (1/2/3)	62/76/4	205/143/6	8.409	0.015
**Insomnia Scores**	2.89 ± 3.70	2.56 ± 3.23	−0.168	0.866
**Insomnia Levels** (No/Subthreshold/Moderate to Severe)	123/17/2	321/28/5	1.250	0.264
**Suicide** (None/Idea without Conduct/Conduct)	109/15/18	272/38/44	0.008	0.996
**MMSE Scores**	25.07 ± 4.12	25.12 ± 4.1	−0.233	0.816
**MMSE Levels** (Normal/Mild/Moderate/Severe)	63/54/25/0	172/131/50/1	0.969	0.325
**PANSS**				
Positive	15.91 ± 5.10	15.74 ± 5.09	−0.472	0.637
Negative	20.51 ± 6.56	20.74 ± 6.20	−0.307	0.759
General	39.02 ± 8.46	38.54 ± 7.89	−0.753	0.452
Total	75.56 ± 16.77	75.01 ± 15.66	−0.405	0.685
**GDS** (No/VeryMild/Mild/Moderate/ModeratelySevere)	37/52/34/18/1	122/137/72/23/0	8.070	0.004
**RBANS**				
Immediate Memory	57.34 ± 15.05	58.31 ± 30.76	−0.458	0.647
Visuospatial/Constructional	79.23 ± 16.56	82.61 ± 18.22	−1.828	0.068
Language	78.63 ± 16.07	82.74 ± 11.24	−1.799	0.072
Attention	79.74 ± 14.92	78.44 ± 15.20	−0.901	0.367
Delayed Memory	65.50 ± 18.55	65.34 ± 19.24	−0.167	0.868
Total	360.49 ± 60.46	369.05 ± 7213	−0.760	0.447

Note: Caries status = patients with DMFT > 0 is defined as “Yes” and DMFT = 0 as “No” ;

DMFT = the total number of decayed, missing, filled teeth; DT = decayed Teeth; FT = filled teeth; GDS = the Global Deterioration Scale; MMSE = Mini-Mental State Examination; MT = missing teeth; PANSS = the Positive and Negative Syndrome Scale; RBANS = Repeatable Battery for the Assessment of Neuropsychological Status;

**Table 2:** Demographic and clinical characteristics between with and without caries grouped by sex in patients with schizophrenia

	Male		Female		Diagnosis		Sex		Diagnosis × Sex		*η* ^2^
	Non-Caries (n = 44)	Caries (n = 310)	Non-Caries (n = 35)	Caries (n = 107)	*F*	*p*	*F*	*p*	*F*	*p*	
**Age** (years)	34.77 ± 8.02	48.96 ± 11.75^**^	34.49 ± 8.29	49.21 ± 10.868^##^	119.519	< 0.001	15.439	< 0.001	10.463	0.001	0.218
**BMI** (kg/m^2^)	24.12 ± 4.15	24.24 ± 3.61	25.06 ± 4.85	25.22 ± 4.33	0.086	0.769	0.389	0.533	0.003	0.953	0.023
**Education Levels** (Primary/Junior/Senior/Bachelor)	3/21/16/0	43/139/97/31	0/22/6/7	10/36/43/18^##^	5.213	0.001	3.498	0.062	4.366	0.005	0.062
**Marriage** (Unmarried/Married/Divorced/Widowed)	34/6/4/0	202/54/52/2	15/12/8/0	49/35/17/2	0.564	0.639	2.378	0.124	0.918	0.432	0.035
**Smoke** (Never/Used/Now)	28/11/5	109/56/145^**^	33/2/0	99/7/1	0.785	0.456	0.034	0.854	0.029	0.971	0.057
**Drinking** (No/Yes)	32/12	210/100	32/3	100/7	0.066	0.797	5.778	0.017	0.430	0.512	0.024
**Diabetes Mellitus** (No/Yes)	44/0	265/45^**^	32/3	80/27^#^	12.358	< 0.001	6.796	0.009	0.215	0.643	0.047
**Hypertension** (No/Yes)	44/0	265/45^**^	31/4	89/18	4.649	0.032	8.601	0.004	0.431	0.517	< 0.001
**Family History** (No/Yes)	37/7	271/39	26/9	79/28	0.094	0.759	5.888	0.016	0.163	0.686	0.023
**First-episode Onset age** (years)	23.41 ± 6.21	25.78 ± 7.71	22.03 ± 4.52	25.81 ± 8.33^#^	12.382	< 0.001	6.137	0.014	2.487	0.115	0.047
**First Hospitalization age** (years)	26.57 ± 7.77	29.66 ± 10.55	22.94 ± 5.01	27.39 ± 9.21^##^	13.306	< 0.001	8.350	0.004	4.185	0.041	0.049
**Illness Duration** (years)	11.36 ± 7.33	23.18 ± 11.56^**^	12.46 ± 8.80	23.40 ± 11.58^##^	69.615	< 0.001	10.226	< 0.001	3.265	0.071	0.149
**Antipsychotics Numbers** (1/2/3)	27/16/1	178/127/5	13/22/0	49/54/4	0.708	0.493	0.131	0.717	1.695	0.185	0.030
**Antipsychotics Type** (Typical/Atypical/Both)	0/39/5	0/301/9**	3/32/0	10/95/2	1.459	0.233	0.006	0.940	3.351	0.068	0.037
**Antipsychotic Dosage** (mg/day)	410.97 ± 838.38	361.68 ± 319.43	244.14 ± 186.45	657.78 ± 1429.23^##^	0.125	0.724	14.707	< 0.001	2.357	0.125	0.032
**Suicide** (None/Idea-without-Conduct/Conduct)	32/7/5	240/31/39	27/3/5	82/12/13	0.037	0.964	3.953	0.047	0.564	0.569	0.026
**Insomnia Scores**	1.89 ± 2.46	2.66 ± 3.32	1.54 ± 2.37	3.33 ± 3.95^##^	10.337	0.001	14.352	< 0.001	2.622	0.106	0.044
**PANSS**											
Positive	15.41 ± 4.51	15.78 ± 5.17	14.46 ± 4.96	16.38 ± 5.08^#^	4.768	0.029	7.291	0.007	3.013	0.083	0.034
Negative	20.91 ± 5.93	20.72 ± 6.24	19.80 ± 5.81	20.74 ± 6.79	0.397	0.529	3.230	0.073	0.682	0.409	0.024
General	39.18 ± 9.16	38.45 ± 7.71	37.20 ± 8.25	39.79 ± 8.43	1.577	0.210	6.343	0.012	3.419	0.065	0.030
Total	75.50 ± 17.40	74.95 ± 15.42	71.46 ± 16.08	76.91 ± 16.85	2.369	0.124	6.000	0.015	3.114	0.078	0.031
**MMSE Levels** (Normal/Mild/Moderate/Severe)	31/10/3/0	141/121/47/1**	15/16/4/0	48/38/21/0	1.274	0.283	10.191	0.002	2.167	0.116	0.044
**GDS** (No/VeryMild/Mild/Moderate/ModeratelySevere)	23/127/7/0	99/125/65/21/0*	11/13/7/4/0	26/39/27/14/1	1.184	0.317	9.907	0.002	0.162	0.923	0.038
**RBANS**											
Immediate Memory	59.44 ± 14.75	57.76 ± 28.96	58.11 ± 14.53	57.80 ± 15.28	0.287	0.592	0.503	0.479	0.070	0.792	0.024
Visuospatial/Constructional	81.32 ± 17.35	81.71 ± 17.92	76.66 ± 15.44	80.07 ± 16.90	0.446	0.505	4.800	0.029	2.309	0.129	0.027
Language	81.76 ± 12.98	81.53 ± 12.93	80.23 ± 15.03	78.11 ± 16.43	0.477	0.490	0.010	0.919	0.256	0.613	0.024
Attention	83.14 ± 14.28	77.99 ± 15.14**	82.71 ± 12.95	78.77 ± 15.44	6.354	0.012	0.350	0.714	0.062	0.803	0.037
Delayed Memory	68.81 ± 18.76	64.74 ± 9.03**	64.31 ± 17.69	65.89 ± 18.89	0.624	0.430	6.504	0.011	2.827	0.093	0.034
Total	370.67 ± 63.82	365.83 ± 70.03	362.03 ± 52.38	359.99 ± 63.09	0.290	0.591	0.576	0.448	0.300	0.862	0.024

Note: DMFT = the total number of decayed, missing, filled teeth; GDS = the Global Deterioration Scale; MMSE = Mini-Mental State Examination; PANSS = the Positive and Negative Syndrome Scale; RBANS = Repeatable Battery for the Assessment of Neuropsychological Status;

* indicates that caries and non-caries groups were significant in male patients. ^*^ p < 0.05; ^**^ p < 0.01

# indicates a significance between caries and non-caries groups in female patients. ^#^ p < 0.05; ^##^ p < 0.01

### Analysis of risk factors for dental caries in male patients

Table 2 shows statistical significance in age (p < 0.001), smoking (p < 0.001), hypertension (p = 0.007), diabetes (p = 0.007), type of antipsychotic drugs (p = 0.007), MMSE level (p = 0.004), GDS ranks (p = 0.040), attention subscale score (p = 0.011), and delayed memory subscale score (p = 0.012) among men between caries groups. Only the comparison of age and smoking passed the Bonferroni test. Supplementary Table 1 shows caries were correlated with age, type of antipsychotic drugs, diabetes, hypertension, smoking status, MMSE levels, GDS ranks, attention scale score, and delayed memory scale score (all p < 0.05), with age, smoking, and illness duration passing the Bonferroni test. Multivariate analysis shows that the independent risk factors for caries are age (p < 0.001, OR = 1.116, 95%CI = 1.074–1.159) and current smoking (p = 0.001, OR = 5.949, 95%CI = 2.049–17.278), adjusted R^2^ = 0.356, as shown in Table 3. In the smoking group, the OR for dental caries was 1.308 (χ^2^ = 0.470, p = 0.493) between previous and non-smoking patients and 5.696 (χ^2^ = 11.610, p = 0.001) between current and previous smoking patients.

**Table 3:** The risk-factor analysis of caries in patients with schizophrenia

		Univariate Analysis				Multivariate Analysis (adjusted)			
		*p*	OR	95% CI		*p*	OR	95%CI	
				Lower	Upper			Lower	Upper
**Male**	Age	< 0.001	1.125	1.084	1.167	< 0.001	1.116	1.074	1.159
	Antipsychotic Type (Atypical)	0.013	4.288	1.367	13.447				
	Diabetes Mellitus (Yes)	0.997	> 1000	0.000					
	Hypertension (Yes)	0.997	> 1000	0.000					
	Smoking Status (Now)	< 0.001	7.450	2.786	19.918	0.001	5.949	2.049	17.278
	MMSE Levels	0.005	2.180	1.271	3.740				
	PANSS								
	Positive	0.647	1.015	0.952	1.083				
	Negative	0.847	0.995	0.946	1.047				
	General	0.562	0.988	0.950	1.028				
	Total	0.826	0.998	0.978	1.018				
	GDS Rank	0.041	1.501	1.016	2.219				
	RBANS								
	Immediate Memory	0.622	0.998	0.990	1.006				
	Visuospatial/Constructional	0.349	0.992	0.975	1.009				
	Language	0.880	0.998	0.970	1.026				
	Attention	0.020	0.974	0.952	0.996				
	Delayed Memory	0.011	0.979	0.963	0.995				
	Total	0.404	0.998	0.994	1.002				
**Female**	Age	< 0.001	1.144	1.090	1.202	< 0.001	1.222	1.132	1.320
	Diabetes Mellitus (Yes)	0.047	3.600	1.020	12.708				
	First-Episode Onset Age	0.014	1.080	1.016	1.148				
	First Hospitalization Age	0.010	1.077	1.018	1.139				
	Illness Duration	< 0.001	1.111	1.059	1.165				
	Total Dosage	0.028	1.002	1.000	1.004	0.029	1.002	1.000	1.004
	Insomnia Scores	0.018	1.216	1.034	1.430				
	PANSS								
	Positive	0.055	1.090	0.998	1.191	0.046	1.106	1.002	1.221
	Negative	0.462	1.023	0.963	1.086				
	General	0.119	1.038	0.990	1.089				
	Total	0.097	1.020	0.996	1.045				
	MMSE Levels	0.424				0.001^*^	0.063	0.012	0.330
	GDS Rank	0.930							
	RBANS								
	Immediate Memory	0.724	0.995	0.971	1.021				
	Visuospatial/Constructional	0.291	1.013	0.989	1.037				
	Language	0.498	0.992	0.968	1.016	0.046	0.959	0.920	0.999
	Attention	0.176	0.982	0.956	1.008				
	Delayed Memory	0.662	1.005	0.984	1.026				
	Total	0.862	0.999	0.993	1.006				

* The significance was found in the mild level of MMSE levels

### Analysis of risk factors for dental caries in female patients

Table 2 shows significant differences in age (p < 0.001), education level (p < 0.001), diabetes (p = 0.036), first-episode onset age (p = 0.031), age at first hospitalization (p = 0.018), the dose of antipsychotic drugs (p = 0.007), insomnia scores (p = 0.007), and PANSS positive scale scores (p = 0.047) among female patients between caries groups. Only age and education level passed the Bonferroni test. Caries were correlated with age, diabetes, age at first-episode onset, age at first hospitalization, insomnia score, the dose of antipsychotic drug, and PANSS positive score (all p < 0.05), as shown in Supplementary Table 1. Multiple regression analysis showed that age, the dose of antipsychotic medications, PANSS positive scores, MMSE level (mild), and language subscale scores were independent risk factors (adjusted R^2^ = 0.587), as shown in Table 3.

## Discussion

Some studies have reported that people with severe mental illness have significantly higher numbers of decayed, missing, or filled teeth than the general population [38]. This study shows similarities and differences in sex-related influential factors for caries between male and female patients with schizophrenia, consistent with our previous research findings [29]. Among them, males are more prone to suffer caries or tooth loss than females, with a higher risk of caries than females. Age is a common risk factor for dental caries in males and females with schizophrenia, which is consistent with the results of Velasco-Ortega et al. [39] and Yang et al. [29]. Caries is correlated with smoking in males with different risk factors for different smoking statuses, while in females, caries is correlated with the dose of antipsychotic medication, PANSS Positive subscale scores, dementia severity (MMSE), or language function subscale scores. The following is an analysis of the relevant factors of dental caries in men and women.

### Common risk characteristics for male and female caries

This study proves that age-induced oral aging is an undeniable natural process [40]. The age-related changes in oral anatomy and function mainly manifest as enamel wear, fragmentation [41], fracture lines, color deposition, and atrophy of pulp chambers and dentinal tubules [42]. At the same time, aging may promote changes in the oral microbial ecology. Previous studies have shown that the diversity of oral bacterial microbiota in healthy Chinese adults is related to sex and age [43]. Further, the abundance and types of oral microbiota in the elderly population may differ from those in young people [44]; for example, there is excessive growth of anaerobic bacteria in the elderly [45, 46]. Changes in the oral flora may trigger inflammatory reactions in the soft and hard tissues of the tooth body [47]. Animal experiments have also confirmed that aging may damage the tooth body and periodontal environment by promoting chronic inflammation, such as interleukin-6 (IL-6) and IL-17, and reducing the ability of dental pulp regeneration [48]. These factors all promote the occurrence of caries with increasing age. In addition, aging cells accumulate in the alveolar bone and promote aging-related secretory phenotypes. At the same time, they have synergistic effects with oral bacteria, destroying hard tooth tissue, causing severe loss of alveolar bone, and aggravation of periodontal inflammation [49]. Oral infectious pathogens, such as cariogenic bacteria, may even cause stroke, diabetes, lower respiratory tract infection, premature delivery, and pneumonia [50, 51], so it is imperative to control oral caries in elderly hospitalized schizophrenic patients.

### Analysis of risk factors for caries due to sex difference

#### Analysis of risk factors of caries in male patients with schizophrenia

This study showed a smoking rate of 59.7% in male patients with schizophrenia, consistent with the rate of 57.5% observed in previous Chinese studies [52]. The high smoking rate in male patients with schizophrenia may be due to several reasons. First, the ward’s social culture, the hospital’s acquiescence, and the difficulty quitting smoking are important factors [53]. Second, nicotine acts on nicotinic acetylcholine receptors by improving neurochemical deficits and thus may help alleviate some symptoms of schizophrenia [54]. Third, there are multiple co-morbid genes between smoking and schizophrenia, and smoking may drive some risk genes for schizophrenia [55]. For example, CHRNA2 is a co-acting target of smoking behavior and schizophrenia [55]. Further, CHRNA5 with variants on chromosome 15q25 was also found to change the daily smoking amount [56]. Smoking alters the composition and diversity of oral microorganisms, increasing cariogenic bacteria, e.g., *Streptococcus mutants* and *Lactobacilli fermentum* [57]. Smoking can also change the abundance of salivary microbes [58] and the associated metabolic functions of the microbial communities [59], leading to a greater susceptibility to dental caries [60]. In addition, poor compliance of psychiatric patients makes caries treatment more difficult.

This study showed no statistically significant difference in caries risk between patients who used to smoke and those who never smoked. In contrast, the OR was 5.7 in current-smoking patients compared to those who used to smoke. Though Velasco-Ortega et al. [39] and Yang et al. [29] both reported that smoking is a risk factor for patients with schizophrenia, they did not further investigate the difference between sex, but our results made up for this deficiency. Our results indicate that smoking cessation reduces the risk of caries and is consistent with previous studies that found that smoking cessation reduces the risk of periodontitis [61] and the rate of tooth loss [62]. After smoking cessation, the altered oral microbial community may explain this, such as the reduced abundance of *Porphyromonas gingivalis*, *Dialister pneumosintes*, and *Treponema denticola*, or the recolonization of beneficial bacteria [63].

#### Analysis of risk factors of caries in female patients with schizophrenia

The results of this study suggest that cognitive impairment (MMSE) in female patients is associated with an increased risk of caries, which is consistent with previous findings [64]. For instance, a correlation was found between patients’ cognitive abilities and dental caries in a study of young and middle-aged adults [65]. Although relatively few studies specifically explored the degree of MMSE and oral hygiene in schizophrenia patients, such studies in elderly Alzheimer’s patients are more comprehensive and in-depth. For example, it has been found that reduced cognitive function in patients may be associated with oral problems such as periodontitis, gingivitis, alveolar bone loss, or attachment loss [66]. Inflammatory factors, such as IL-1β and tumor necrosis factor-alpha (TNF-α), are the first to be upregulated when periodontal inflammation occurs, and they may promote a pro-inflammatory environment in the brain that leads to the development of cognitive dysfunction [67]. Experiments in mice have found that saliva-associated oral microorganisms can exacerbate dementia symptoms through the gut-brain axis [68]. In addition, *Porphyromonas gingivalis* actively invaded the brains of mice during infection [69], leading to beta-amyloid deposition and causing cognitive impairment [67]. This point may be an initiating factor for the reduced delayed verbal recall function in dementia patients [70]. Clinical postmortem reports confirmed the presence of *Porphyromonas gingivalis* in the brain of Alzheimer’s patients [71], suggesting that oral microorganisms may have the ability to influence cognition in patients with dementia. The presence of oral microorganisms represented by *Porphyromonas gingivalis* may be associated with cognitive impairment, which may also explain the finding of cognitive alteration in patients with caries in our study. In other words, the disturbed oral ecology of patients with schizophrenia may further contribute to their cognitive impairment and the increased risk of developing dental caries.

Previous studies have shown an independent correlation between PANSS-positive symptoms and the number of lost teeth [72], and positive symptoms also increase the risk of caries [73]. These conclusions may suggest that the increase in positive symptoms will increase caries risk in female patients in this study. Positive symptoms, including hallucinations and delusions, as the main symptoms of schizophrenia, may have different effects on the onset and development of dental caries, so in-depth research is needed.

The results of this study indicate that an increase in the dosage of antipsychotic drugs in female patients with schizophrenia leads to an increased risk of dental caries since most antipsychotic drugs are bacterial antagonists [74], which may lead to a disruption of the patient’s oral or gut microbiota, particularly in women [75]. Long-term and high-dose use of antipsychotic medication in patients with schizophrenia may lead to the emergence of cariogenic bacterial resistance, such as a noticeable increase in gut lactobacilli levels after 24 weeks of risperidone treatment in first-episode schizophrenia patients [76]. Antipsychotic drugs can increase prolactin levels, and the degradation of prolactin inducible protein is correlated with caries [77]. In addition, antipsychotic drugs may reduce the number of white blood cells [78], disrupt the endocrine system [79], and lead to vitamin deficiency, reducing the immune response of the oral cavity and leaving the body susceptible to pathogenic microorganisms. At the same time, antipsychotic drugs can cause dry mouth [80, 81] and affect the oral mucosal barrier effect. Long-term use of high-dose antipsychotic drugs may exacerbate the adverse reactions mentioned above [82], leading to an increased risk of caries in patients.

Above all, these differences in patients with schizophrenia make it necessary to incorporate oral hygiene monitoring into routine management to improve the treatment approach and outcomes for patients with schizophrenia. Oral hygiene in patients with schizophrenia and the corresponding microbial-gut-brain axis-related mechanisms have become a hot topic, and studies on caries-based microbial oral hygiene problems may improve clinical symptom treatment and prognosis in patients with schizophrenia. Therefore, this study of sex differences in caries in patients with schizophrenia provides evidence for the individualized treatment of the disease.

### Limitations

There were several limitations which should be concerned. Firstly, our study included hospitalized patients with a long course of illness and long-term use of antipsychotic drugs. The results of this study cannot be generalized to other types of patients, such as outpatient and community patients. Secondly, due to the different mechanisms of action, the oral effects of antipsychotic drugs may vary. Thirdly, the sample balance between sex is insufficient, and the sample size of included patients needs to be expanded. Fourthly, the data about antiparkinsonian agents had not been collected, since they may also affect caries development due to dry mouth. Finally, due to the cross-sectional nature of this survey, we cannot determine whether a causal relationship exists between clinical factors and dental caries. Further prospective research is needed to analyze the pathogenic factors of dental caries.

## Conclusions

The risk of dental caries and caries index in males was higher than in females among hospitalized patients with schizophrenia, indicating that the oral hygiene status of male schizophrenia patients may be worse. This report has shown that age is a common risk factor for male and female caries. The risk of dental caries in male patients with schizophrenia is associated with smoking, general symptoms, BMI, and types of antipsychotic drugs. In contrast, in females, it is correlated to the dosage of antipsychotic medications used, positive symptoms, and degree of dementia. Therefore, to address the different caries risk factors brought about by sex differences, further prevention and treatment plans should be formulated. By regulating the oral microbiota and leveraging the new mechanism of the “brain-gut axis” pathway, new ideas and methods for diagnosing and treating schizophrenia can be developed.

### Electronic supplementary material

Below is the link to the electronic supplementary material.

Supplementary Material 1: Supplementary Table 1. The correlation between risk factors and caries in male and female patients with schizophrenia.

## Data Availability

The datasets generated and/or analyzed during the current study are not publicly available due but are available from the corresponding author on reasonable request.
