# Supplementary material for: Sex-based influential factors for dental caries in patients with schizophrenia
Source: BMC Psychiatry. 2023 Oct 10;23:735. doi: 10.1186/s12888-023-05256-z (PMC10566046; doi:10.1186/s12888-023-05256-z)
Supplement: Supplementary file 1 — Supplementary Material 1: Supplementary Table 1. The correlation between risk factors and caries in male and female patients with schizophrenia. [file 12888_2023_5256_MOESM1_ESM.docx]

| Supplementary Table 1. The correlation between risk factors and caries in male and female patients with schizophrenia. | | | | |
| --- | --- | --- | --- | --- |
|  | Man | | Woman | |
|  | *rho* | p | *rho* | p |
| Age | 0.377 | <0.001 | 0.519 | <0.001 |
| BMI | 0.022 | 0.682 | 0.058 | 0.491 |
| Education levels | −0.042 | 0.426 | 0.070 | 0.407 |
| Marriage | 0.089 | 0.095 | −0.014 | 0.867 |
| Diabetes Mellitus | 0.144 | 0.007 | 0.176 | 0.036 |
| Hypertension | 0.144 | 0.007 | 0.064 | 0.448 |
| Family History | −0.033 | 0.540 | 0.004 | 0.958 |
| Drinking Status | 0.035 | 0.507 | −0.034 | 0.686 |
| Smoking Status | 0.235 | <0.001 | 0.031 | 0.718 |
| First-Episode Onset Age | 0.103 | 0.052 | 0.181 | 0.031 |
| First Hospitalization Age | 0.081 | 0.128 | 0.198 | 0.018 |
| Illness Duration | 0.336 | <0.001 | 0.425 | <0.001 |
| Antipsychotics Numbers | 0.024 | 0.654 | −0.054 | 0.525 |
| Antipsychotics Type | 0.143 | 0.007 | 0.013 | 0.877 |
| Total Dosage (mg) | 0.103 | 0.053 | 0.228 | 0.006 |
| Insomnia Levels | 0.034 | 0.528 | 0.130 | 0.123 |
| Suicide Status | −0.030 | 0.578 | −0.001 | 0.995 |
| PANSS |  |  |  |  |
| Positive | 0.010 | 0.849 | 0.167 | 0.046 |
| Negative | −0.022 | 0.675 | 0.035 | 0.682 |
| General | −0.014 | 0.790 | 0.125 | 0.137 |
| Total | −0.001 | 0.991 | 0.141 | 0.095 |
| MMSE Scores | −0.158 | 0.003 | −0.036 | 0.667 |
| MMSE Levels | 0.162 | 0.002 | 0.024 | 0.781 |
| GDS Rank | 0.120 | 0.024 | 0.080 | 0.343 |
| RBANS |  |  |  |  |
| Immediate Memory | −0.090 | 0.090 | −0.039 | 0.645 |
| Visuospatial/Constructional | −0.054 | 0.309 | 0.072 | 0.391 |
| Language | 0.040 | 0.452 | −0.059 | 0.483 |
| Attention | −0.135 | 0.011 | −0.105 | 0.213 |
| Delayed Memory | −0.134 | 0.011 | 0.022 | 0.795 |
| Total | −0.095 | 0.076 | −0.051 | 0.545 |
|  | | |  |  |
